# Prognostic Role of Tumor-Infiltrating Lymphocytes in Oral Squamous Cell Carcinoma

**DOI:** 10.1186/s12885-024-12539-5

**Published:** 2024-06-26

**Authors:** Wattawan Wongpattaraworakul, Allen Choi, Marisa R. Buchakjian, Emily A. Lanzel, Anand Rajan KD, Andrean L. Simons

**Affiliations:** 1 Department of Oral Pathology, Radiology, and Medicine, College of Dentistry, University of Iowa, Iowa City, IA United States; 2 Department of Pathology, College of Medicine, University of Iowa Hospitals and Clinics, Iowa City, IA United States; 3 Department of Otolaryngology – Head and Neck Surgery, University of Iowa Hospitals and Clinics, Iowa City, IA United States; 4 Holden Comprehensive Cancer Center, University of Iowa Hospitals and Clinics, Iowa City, IA United States; 5 Department of Radiation Oncology, University of Iowa Hospitals and Clinics, B180K Medical Laboratories Iowa City, IA 52242 Iowa City, United States

**Keywords:** OSCC, CD3, CD4, CD8, TIL, TMA

## Abstract

**Background:**

In oral squamous cell carcinoma (OSCC), the tumor-node-metastasis (TNM) staging system is a significant factor that influences prognosis and treatment decisions for OSCC patients. Unfortunately, TNM staging does not consistently predict patient prognosis and patients with identical clinicopathological characteristics may have vastly different survival outcomes. Host immunity plays an important role in tumor progression but is not included in the TNM staging system. Tumor-infiltrating lymphocytes (TILs) are part of the host immune response that recognizes tumor cells; and the presence of TILs has emerged as potential candidates for prognostic markers for many types of cancers. The present study aims to determine the association of T cell-specific markers (CD3, CD4, CD8, and FOXP3) with clinicopathological characteristics and survival outcomes in OSCC patients. The prognostic value of CD3, CD4, and CD8 will also be evaluated based on tumor stage.

**Methods:**

Tissue microarrays were constructed containing 231 OSCC cases and analyzed by immunohistochemical staining for the expression of CD3, CD4, CD8, and FOXP3. The expression scores for each marker were correlated with clinicopathological parameters and survival outcomes. The prognostic impact of CD3, CD4 and CD8 were further analyzed based on tumor stage (early or advanced).

**Results:**

CD3, CD4, and CD8 were found to be significantly associated with both overall survival and progression-free survival using univariate analysis. However, none of these markers were found to independently predict the survival outcomes of OSCC using multivariate analysis. Only conventional factors such as nodal status, tumor differentiation and perineural invasion (PNI) were independent predictors of survival outcomes, with nodal status being the strongest independent predictor. Additionally, low CD4 (but not CD3 or CD8) expression was found to identify early-stage OSCC patients with exceptionally poor prognosis which was similar to that of advanced staged OSCC patients.

**Conclusions:**

TIL markers such as CD3, CD4, CD8, and FOXP3 can predict the survival outcomes of OSCC patients, but do not serve as independent prognostic markers as found with conventional factors (i.e. nodal status, tumor differentiation and PNI). CD4 expression may assist with risk stratification in early-stage OSCC patients which may influence treatment planning and decision making for early-stage OSCC patients.

**Supplementary Information:**

The online version contains supplementary material available at 10.1186/s12885-024-12539-5.

## Introduction

Among all malignancies in the oral cavity, oral squamous cell carcinoma (OSCC) accounts for 90% of these malignancies [1, 2]. OSCC is associated with risk-factors such as chronic tobacco smoking, smokeless tobacco alcohol intake, and other factors including genetic predisposition and human papillomavirus virus (HPV) infection [1–3]. In parts of Asia and the Pacific, areca (betel) nut chewing is a high-risk factor for the development of oral submucous fibrosis, the oral lesion most strongly linked to OSCC [1–3]. OSCCs can arise in the alveolar ridge, buccal mucosa, floor of the mouth, palate, tongue and other locations within the oral cavity [4]. The standard treatment approaches for OSCC are surgery, radiotherapy, chemotherapy, or a combination of these strategies depending on co-morbidity factors and the extent of the disease. However, OSCC patients have a high risk of treatment failure and the main disease-related mortality in OSCC patients is due to locoregional recurrence [5]. In fact, the survival of OSCC patients remains poor (~ 50%) and relatively unchanged for the last several decades [6].

Treatment decisions depend primarily on the TNM classification system where “T” considers the tumor size, “N” considers the presence of nodal metastasis; and “M” considers the presence of distant metastases. The TNM staging has been reported as a significant factor that influences the prognosis of OSCC patients, with lymph nodal metastasis (N) being the strongest independent predictor of worse outcomes without disseminating disease. OSCCs detected in the early stages are expected to have a favorable prognosis, typically managed by surgery [7, 8]. Elective neck dissection or post-operative radiotherapy might be given in cases with other unfavorable characteristics, such as the presence of poor differentiation, perineural invasion (PNI), or lymphovascular invasion (LVI) [9]. Unfortunately, TNM staging does not consistently predict patient prognosis [6]. For example, a subset of early-stage OSCCs may experience disease progression and require additional adjunct therapies [8]. Identification of these early stage OSCC patients is crucial, as more aggressive or alternative treatment may be necessary, and a delay in appropriate treatment may worsen the prognosis [10]. Therefore, there is a need for more accurate prognostic indicators that may complement the TNM staging system to guide treatment decisions in a timely manner for OSCC patients.

Host immunity plays a complex but important role in tumor progression but is not included in the TNM staging system. Tumor-infiltrating lymphocytes (TILs) are part of the immune response that plays a critical role in recognizing and eliminating tumors [11]. Previous work has shown that the presence of TILs can significantly affect patient survival in several types of malignancies. Hence the study of TILs has emerged as a potential prognostic candidate for various types of cancers [11–14]. The majority of TILs are T cells, which can be detected using the CD3 marker. Among T-cells, there are subpopulations that includes cytotoxic T-cells, T-helper cells, and regulatory T-cells (Treg) [15, 16]. Cytotoxic T-cells, detected by the CD8 marker, are particularly important in their function against cancer cells. CD8 + T cells can directly kill cancer cells and secrete tumoricidal cytokines to help eradicate tumors [17–20]. T-helper cells play a crucial role in supporting the function of cytotoxic T-cells through several mechanisms. These cells secrete cytokines that promote the differentiation and proliferation of cytotoxic T-cells. Additionally, they can secrete tumoricidal cytokines in response to dendritic cells [21]. The CD4 marker is used to identify T-helper cells, which are the most frequent subtype of CD4 + T-cells. Tregs can also be detected by CD4 markers, as well as by the presence of the FOXP3 protein [20, 21]. Tregs secrete suppressive cytokines that limit the anti-tumor immune response by preventing the expansion of tumoricidal cytokines, suppressing the function of effector T-cells, and inhibiting the maturation of dendritic cells [22, 23].

Previous biomarker studies of OSCCs with TIL marker expression are contradictory, where some have shown that high expression of select TIL markers (e.g. CD3, CD4, FOXP3 and CD8) are associated with favorable patient clinicopathological characteristics and survival outcomes; and others showing the opposite result or no association with survival outcomes [24–27]. Additionally, there are a lack of studies examining the role of TIL marker expression in tumor recurrence risk assessment in early-stage disease. In this study, we aim to investigate a cohort of OSCC patients to (i) determine the prognostic value of T cell-specific markers (CD3, CD4, CD8, and FOXP3) by scoring them using categorical variables, and (ii) determine whether the inclusion of T cell-specific markers (CD3, CD4, and CD8) would identify early-stage OSCC patients with a high risk of tumor recurrence and treatment failure.

## Materials and methods

### Tissue Microarrays (TMAs)

231 formalin-fixed paraffin-embedded specimens of OSCC resections/biopsies were obtained from the University of Iowa Hospital and Clinics and the College of Dentistry (University of Iowa) between 2004–2014. Tissue slides were reviewed to confirm the original diagnosis, and clinicopathological characteristics were collected, including age, sex, smoking history, tumor location, T-stage, N-stage, tumor differentiation, PNI, LVI, and bone invasion. The clinicopathological characteristics were obtained from medical records where tumor microscopic features and TNM stage had been previously evaluated by board-certified pathologists, some of which were based on the American Joint Committee on Cancer 7 (AJCC7). The TNM status was re-evaluated based on the updated version of AJCC8. According to AJCC8, patients who have not received regional lymph node dissection and have no evidence of palpable or radiographically suspicious lymph node metastasis are designated as having clinical N0. Patients were also subclassified based on the stage of OSCC. Patients who have T1 and T2 OSCCs with no lymph node metastasis were designated as early-stage OSCC patients. Patients who have T3 and T4 OSCC, as well as those who have any lymph node metastasis with any T-stage OSCCs, were designated as advanced-stage OSCC patients. TMAs were constructed using at least 3 representative areas per case (1 case with 2 representative areas). Information on local recurrence and distant metastasis was collected from patients who followed up at the University of Iowa Hospital and Clinics until a recent date or until death in addition to information from the Iowa Cancer Registry. The lack of clinicopathological characteristics information was due to information that was not provided from medical charts. Fifty percent of patients underwent surgery as a single therapy while the other 50% of patients underwent surgery with or without chemotherapy.

### Immunohistochemistry (IHC)

Antigen retrieval was performed on freshly cut sections using a decloaking chamber for 5 min at 125 °C in TRIS buffer (pH 9.0). Endogenous peroxidase was blocked by incubation with 3% peroxide at room temperature for 8 min. IHC was performed using the following antibodies:

- Pan T-cells were detected with a CD3 antibody (Dako A0452) at a 1:200 dilution.
- T-helper cells were detected with a CD4 antibody (Novocastra NCL-L-CD4-368) at a 1:100 dilution.
- Cytotoxic T cells were detected with a CD8 antibody (Dako M7103) at a 1:100 dilution.
- T regulatory cells (Tregs) were detected with a FOXP3 antibody (Thermo Fisher Scientific 14–4777-82) at a 1:100 dilution.

### Evaluation of TIL staining

**Fig. 1:**
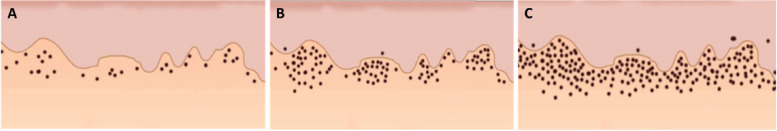
Semiquantitative grading based on the density of positive inflammatory cells. Example images represent single isolated positive cells or small aggregates of positive cells (2–4 cells) (**A**); discrete nodules or aggregates of positive cells with more than 2–4 cells (**B**); and bands or continuous aggregates of positive cells (**C**)

CD3, CD4, CD8, and FOXP3 were evaluated based on the density of positive inflammatory cells using a scoring system from 0 to 3. A score of 0 represents no or very few positive cells; a score of 1 represents single isolated positive cell or small aggregates of positive cells with 2–4 cells; a score of 2 represents discrete nodules or aggregates of positive cells with more than 2–4 cells; and a score of 3 represents bands or continuous aggregates of positive cells (Fig. 1). Patients with immunoscores of 3 did not show significantly better survival outcomes (overall survival [OS], progression-free survival [PFS]) than those with immunoscores of 2 (SupplementalFig. 1–3); therefore, cases with immunoscores of 3 or 2 were combined and designated as high expression ( +). Similarly, patients with immunoscores of 1 did not exhibit significantly better survival than those with immunoscores of 0 (SupplementalFig. 1–3); therefore, immunoscores of 1 or 0 were combined and designated as low expression (-).

### Statistical analysis

The presence of TILs was evaluated using categorical variables; as representing TILs as categorical variables facilitates easier clinical implementation compared to continuous variables [28]. The association between immunostaining scores and patients' clinicopathological characteristics was analyzed using the Chi-square test. Survival outcome differences were plotted using the Kaplan–Meier method and hazard ratios were estimated using Cox proportional hazards modeling. OS was defined as the length of time (in months) from the date of surgery to the date of death from any cause, while PFS was defined as the time from surgery to disease progression or death (in months) from any cause. Differences between survival curves were compared using the Log-rank (Mantel-Cox) test. An alternative test of Gehan-Breslow-Wilcoxon was also performed. Patients (*n* = 7) who followed up at the University of Iowa Hospital and Clinics for less than 1 month or died within 1 month were excluded from the analysis of PFS. Subgroup analysis based on tumor clinical stage was also performed. All testing was performed using GraphPad Prism 9 and R 4.2.2.

## Results

### Clinicopathological characteristics

Table 1 shows the clinicopathological characteristics of the OSCC patient cohort. Of the 231 patients, 59% were male with an average age of 60 years; and 41% were female with an average age of 65 years. The majority of patients were active smokers (49%) at the time of diagnosis, 27% of patients had a history of smoking but had quit, and 24% were non-smokers (Table 1). The most common oral cavity sublocation was the tongue (40%), followed by the floor of mouth (FOM, 13%) and gingiva (13%). A third of the patients had cancer on overlapping sites or other locations such as buccal mucosa, retromolar region, and hard palate. Tumors were mostly moderately differentiated (64%) and without PNI (55%), LVI (68%), or bone invasion (BI, 70%). Only 3 patients initially presented with metastasis. Additionally, tumors were mostly advanced stage (60%), T3/T4 (46%), and N0 (62%). Thirty percent of patients experienced a local recurrence, and 20% experienced distant metastases (Table 1). Significantly worse survival outcomes (OS and PFS) were observed in patients bearing tumors that were high T stage (T3/T4), advanced stage and poorly differentiated along with the presence of lymph node metastasis, PNI, LVI and BI (SupplementalFig. 4–6). Clinicopathological characteristics were also analyzed according to the 3 major oral cavity sublocations (tongue, FOM, and gingiva) (Table 2). FOM tumors occurred in mostly males (84%) compared to females (16%, *p* = 0.007) and were significantly associated with active smoking (*p* < 0.001), advanced stage (*p* = 0.02) and T3/T4 stage (*p* < 0.001) (Table 2). Gingiva tumors were significantly associated with increased age at diagnosis (*p* = 0.007), advanced (*p* = 0.02) and T3/T4 stage (*p* < 0.001) and the lack of PNI (*p* = 0.02) (Table 2).

**Table 1:** Clinicopathological Characteristicsof OSCC Patients

Characteristics	Total Patients n (%)
Total Patients = 231	
**Sex**	
Male	136 (58.9%)
Female	95 (41.1%)
Age Average [± stdev]*	
Male	60.1 [11.8]
Female	64.7 [16.4]
Smoking History	
Active smoker	113 (49.1%)
Never smoker	56 (24.4%)
Quit < 10 Years	14 (6.1%)
Quit > 10 Years	47 (20.4%)
Tumor Site	
Tongue	93 (40.3%)
Floor of mouth	31 (13.4%)
Gingiva	30 (13%)
Overlapping sites	46 (19.9%)
Other sites	31 (13.4%)
T Stage	
T1	70 (31.4%)
T2	50 (22.4%)
T3/T4	103 (46.2%)
N Stage	
Negative	144 (62.3%)
Positive	87 (37.7%)
Stage	
Early	89 (39.6%)
Advanced	136 (60.4%)
Differentiation	
Well	34 (14.9%)
Moderate	146 (64%)
Poor	48 (21.1%)
Perineural invasion	
Yes	99 (44.8%)
No	122 (55.2%)
Lymphovascular invasion	
Yes	71 (32%)
No	151 (68%)
Bone invasion	
Yes	70 (30.3%)
No	161 (69.7%)
Local recurrence	
Yes	50 (29.9%)
No	117 (70.1%)
Distant metastasis	
Yes	33 (19.8%)
No	134 (80.2%)

^*a*^Average age at diagnosis

**Table 2:** Clinicopathological Characteristics According to OSCC Sublocation

	**OSCC Sublocation n (%)**			
Characteristics	**Tongue** **(*n*** **= 93)**	**Floor of mouth** **(*n* = 31)**	**Gingiva** **(*n*** **= 30)**	***p*-value**
Sex				
Male	50 (53.8%)	26 (83.9%)	15 (50%)	0.007
Female	43 (46.2%)	5 (16.1%)	15 (50%)	
Age*				
Average [± stdev]	58.6 [15.5]	58.9 [10.1]	71.4 [10.1]	< 0.001
Smoking history				
Active smoker	39 (42.4%)	26 (83.9%)	8 (26.7%)	< 0.001
Never smoker	22 (23.9%)	3 (9.7%)	9 (30%)	
Former smoker	31 (33.7%)	2 (6.5%)	13 (43.3%)	
T stage				
T1	38 (42.2%)	7 (23.3%)	9 (33.3%)	< 0.001
T2	29 (32.2%)	8 (26.7%)	0 (0%)	
T3/T4	23 (25.6%)	15 (50%)	18 (66.7%)	
N stage				
No	66 (71%)	20 (64.5%)	22 (73.3%)	0.72
Yes	27 (29%)	11 (35.5%)	8 (26.7%)	
Stage				
Early stage	51 (56.7%)	11 (35.5%)	9 (32.1%)	0.02
Advanced stage	39 (43.3%)	20 (64.5%)	19 (67.9%)	
Differentiation				
Well	20 (21.7%)	3 (9.7%)	8 (27.6%)	0.28
Moderate	53 (57.6%)	24 (77.4%)	16 (55.2%)	
﻿Poorly	19 (20.7%)	4 (12.9%)	5 (17.2%)	
Perineural invasion				
Yes	44 (48.9%)	14 (45.2%)	5 (18.5%)	0.02
No	46 (51.1%)	17 (54.8%)	22 (81.5%)	
Lymphovascular invasion				
Yes	22 (24.2%)	9 (29%)	9 (32.1%)	0.67
No	69 (75.8%)	22 (71%)	19 (67.9%)	
Local recurrence				
Yes	21 (30.9%)	4 (22.2%)	5 (22.7%)	0.64
No	47 (69.1%)	14 (77.8%)	17 (77.3%)	
Distance metastasis				
Yes	11 (16.7%)	4 (23.5%)	0 (0%)	0.21
No	55 (83.3%)	13 (76.5%)	22 (100%)	

^*a*^Average age at diagnosis

### Clinicopathological characteristics based on TIL Status

The expression of the TIL markers CD3, CD4, CD8 and FOXP3 were analyzed for associations with clinicopathological characteristics. CD3- and CD4- tumors were significantly associated with advanced stage (*p* < 0.001), high T stage (T3/T4, (*p* < 0.001)), positive lymph node metastasis (*p* < 0.001), poor tumor differentiation (*p* = 0.004) and presence of PNI (CD3: *p* = 0.006, CD4: *p* = 0.001), LVI (CD3: *p* = 0.02, CD4: *p* < 0.001) and BI (CD3: *p* < 0.001, CD4: *p* = 0.02) compared to CD3 + and CD4 + tumors respectively (Table 3). CD8- tumors were significantly associated with the same clinicopathological characteristics as CD3- and CD4- tumors except LVI (p = 0.19). CD8- tumors were also associated with younger age at diagnosis (p = 0.03, Table 3). FOXP3- tumors were significantly associated with male patients (*p* = 0.007), advanced (*p* = 0.005) and high T-stage (*p* = 0.01), and positive lymph node metastasis (*p* = 0.006) (Table 3**).** When TIL expression was analyzed in the 3 major oral cavity sublocations (tongue, floor of mouth and gingiva), we found that FOM tumors were significantly associated with CD3- (*p* = 0.01), CD4- (*p* = 0.01), and FOXP3- expression (*p* = 0.04) (Table 4).

**Table 3:** Clinicopathological Characteristics based on TIL Status

	**CD3**			**CD4**			**CD8**			**FOXP3**		
Characteristic n (%)	**CD3 + ** **(*n*** **= 89)**	**CD3-** **(*n*** **= 138)**	***p*-value**	**CD4 + ** **(*n*** **= 96)**	**CD4-** **(*n*** **= 134)**	***p*-value**	**CD8 + ** **(*n*** **= 49)**	**CD8-** **(*n*** **= 178)**	***p*-value**	**FOXP3 + ** **(*n*** **= 28)**	**FOXP3-** **(*n*** **= 198)**	***p*-value**
Sex												
Male	50 (56.2%)	83 (60.1%)	0.55	53 (55.2%)	83 (61.9%)	0.31	24 (49%)	109 (61.2%)	0.12	10 (35.7%)	124 (62.6%)	0.007
Female	39 (43.8%)	55 (39.9%)		43 (44.8%)	51 (38.1%)		25 (51%)	69 (38.8%)		18 (64.3%)	74 (37.4%)	
Age*												
Avg [± stdev]	60.9 [16]	62.9 [12.5]	0.32	62.4 [14.8]	61.9 [13.4]	0.77	65.8 [13.4]	61 [14.2]	0.03	65 [16.1]	61.6 [13.5]	0.29
Smoking history												
Active	43 (48.9%)	69 (50%)	0.99	45 (47.4%)	68 (50.8%)	0.88	20 (40.8%)	91 (51.4%)	0.37	9 (32.1%)	104 (52.8%)	0.08
Never	22 (25%)	34 (24.6%)		24 (25.3%)	32 (23.9%)		15 (30.6%)	40 (22.6%)		11 (39.3%)	44 (22.3%)	
Former	23 (39.7%)	35 (60.3%)		26 (27.4%)	34 (25.4%)		14 (28.6%)	46 (26%)		8 (28.4%)	49 (24.9%)	
T Stage												
T1	42 (50%)	26 (19.3%)	< 0.001	42 (46.2%)	28 (21.4%)	< 0.001	26 (56.5%)	43 (24.9%)	< 0.001	15 (57.7%)	55 (28.7%)	0.01
T2	18 (21.4%)	31 (23%)		21 (23.1%)	29 (22.1%)		8 (17.4%)	41 (23.7%)		4 (15.4%)	45 (23.4%)	
T3/T4	24 (28.6%)	78 (57.8%)		28 (30.8%)	74 (56.5%)		12 (26.1%)	89 (51.4%)		7 (26.9%)	92 (47.9%)	
N Stage												
No	74 (83.2%)	68 (49.3%)	< 0.001	80 (83.3%)	63 (47%)	< 0.001	38 (77.5%)	105 (59%)	0.02	24 (85.7%)	117 (59.1%)	0.006
Yes	15 (16.8%)	70 (50.7%)		16 (16.7%)	71 (53%)		11 (22.5%)	73 (41%)		4 (14.3%)	81 (40.9%)	
Stage												
Early	53 (63.1%)	35 (25.5%)	< 0.001	56 (61.5%)	33 (24.8%)	< 0.001	28 (60.9%)	61 (34.9%)	0.001	17 (65.4%)	71 (36.6%)	0.005
Advanced	31 (36.9%)	102 (74.5%)		35 (38.5%)	100 (75.2%)		18 (39.1%)	114 (65.1%)		9 (34.6%)	123 (63.4%)	
Differentiation												
Well	23 (26.4%)	17 (12.4%)	0.004	24 (25.5%)	16 (12%)	0.004	14 (29.8%)	26 (14.7%)	0.02	7 (26.9%)	33 (16.7%)	0.4
Moderate	54 (62.1%)	85 (62%)		58 (61.7%)	81 (60.9%)		28 (59.6%)	109 (61.6%)		15 (57.7%)	122 (61.6%)	
Poorly	10 (11.5%)	35 (25.6%)		12 (12.8%)	36 (27.1%)		5 (10.6%)	42 (23.7%)		4 (15.4%)	43 (21.7%)	
Perineural invasion												
Yes	28 (33.73%)	71 (53%)	0.006	28 (31.5%)	70 (53.4%)	0.001	13 (28.9%)	86 (50%)	0.01	8 (32%)	87 (45.5%)	0.2
No	55 (66.27%)	63 (47%)		61 (68.5%)	61 (46.6%)		32 (71.1%)	86 (50%)		17 (68%)	104 (54.5%)	
Lymphovascular invasion												
Yes	19 (22.9%)	52 (38.5%)	0.02	16 (17.8%)	55 (42%)	< 0.001	11 (24.4%)	60 (34.7%)	0.19	5 (19.2%)	64 (33.5%)	0.14
No	64 (77.1%)	83 (61.5%)		74 (82.2%)	76 (58%)		34 (75.6%)	113 (65.3%)		21 (80.8%)	127 (66.5%)	
Bone invasion												
Yes	15 (16.85%)	55 (39.86%)	< 0.001	21 (21.9%)	49 (36.6%)	0.02	9 (18.4%)	59 (33.2%)	0.046	6 (21.4%)	62 (31.3%)	0.29
No	74 (83.15%)	83 (60.14%)		75 (78.1%)	85 (63.4%)		40 (81.6%)	119 (66.8%)		22 (78.6%)	136 (68.7%)	
Local recurrence												
Yes	19 (28.8%)	29 (29.3%)	0.94	18 (25.7%)	32 (33.3%)	0.29	9 (22.5%)	40 (32%)	0.25	5 (23.8%)	45 (31.7%)	0.46
No	47 (71.2%)	70 (70.7%)		52 (74.3%)	64 (66.7%)		31 (77.5%)	85 (68%)		16 (76.2%)	97 (68.3%)	
Distant metastasis												
Yes	9 (14.1%)	24 (23.8%)	0.13	9 (13%)	24 (24.7%)	0.06	7 (17.5%)	25 (20%)	0.73	3 (14.3%)	28 (19.7%)	0.55
No	55 (85.9%)	77 (76.2%)		60 (87%)	73 (75.3%)		33 (82.5%)	100 (80%)		18 (85.7%)	114 (80.3%)	

^*^Average age at diagnosis

**Table 4:** TIL Status Based on OSCC Sublocation

	**Tongue** **(*n*** **= 93)**	**Floor of mouth** **(*n*** **= 31)**	**Gingiva** **(*n*** **= 30)**	***p*-value**
CD3				
High	49 (55.1%)	8 (25.8%)	12 (40%)	0.01
Low	40 (44.9%)	23 (74.2%)	18 (60%)	
CD8				
High	23 (25%)	2 (6.7%)	7 (23.3%)	0.1
Low	69 (75%)	28 (93.3%)	23 (76.7%)	
CD4				
High	49 (53.3%)	7 (22.6%)	16 (53.3%)	0.01
Low	43 (46.7%)	24 (77.4%)	14 (46.7%)	
FOXP3				
High	15 (16.7%)	1 (3.2%)	8 (26.7%)	0.04
Low	75 (83.3%)	30 (96.8%)	22 (73.3%)	

### Prognostic impact by clinicopathological characteristics and TIL expression

**Fig. 2:**
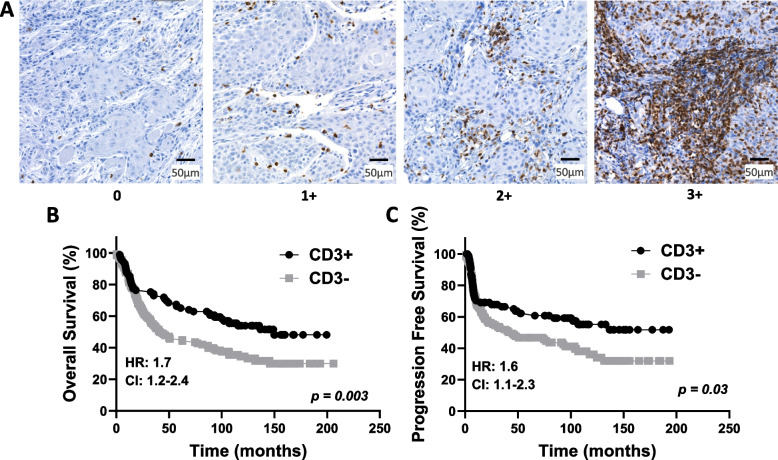
Prognostic Impact of CD3 Expression in OSCC. **A** Shown are images of negative [0], low [1 +], moderate [2 +], and strong [3 +] CD3 expression scores. **B**-**C** Shown are Kaplan–Meier estimates of Overall Survival (**B**) and Progression Free Survival (**C**) according to CD3 + (immunoscores of 2 + and 3 +) and CD3- (immunoscores of 0 and 1 +) in OSCC patients. HR: hazard ratio estimated using Cox proportional hazards modeling. CI: 95% confidence interval

**Fig. 3:**
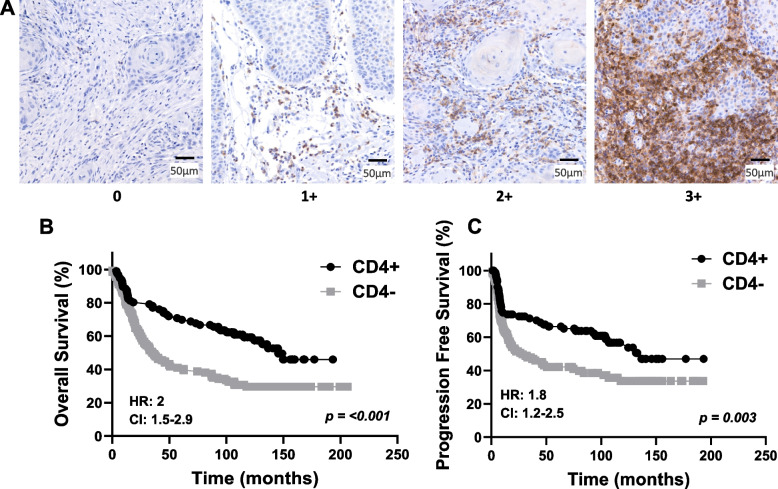
Prognostic Impact of CD4 Expression in OSCC. **A** Shown are images of negative [0], low [1 +], moderate [2 +], and strong [3 +] CD4 expression scores. **B**-**C** Shown are Kaplan–Meier estimates of Overall Survival (**B**) and Progression Free Survival (**C**) according to CD4 + (immunoscores of 2 + and 3 +) and CD4- (immunoscores of 0 and 1 +) in OSCC patients. HR: hazard ratio estimated using Cox proportional hazards modeling. CI: 95% confidence interval

**Fig. 4:**
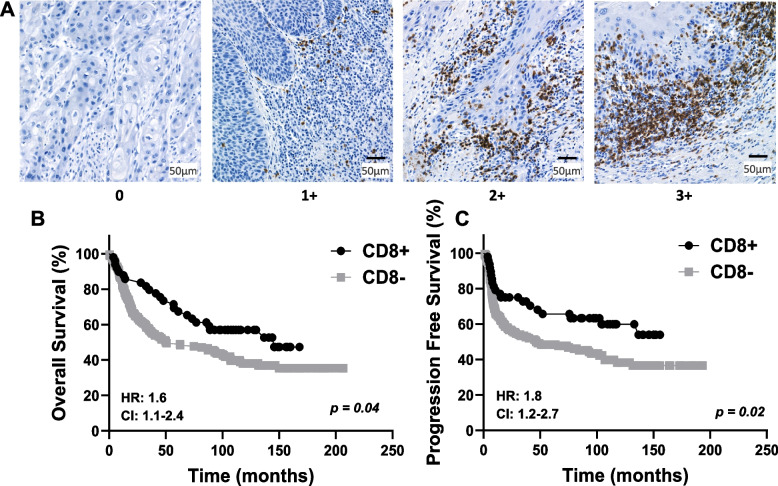
Prognostic Impact of CD8 Expression in OSCC. **A** Shown are images of negative [0], low [1 +], moderate [2 +], and strong [3 +] CD8 expression scores. B-C: Shown are Kaplan–Meier estimates of Overall Survival (**B**) and Progression Free Survival (**C**) according to CD8 + (immunoscores of 2 + and 3 +) and CD8- (immunoscores of 0 and 1 +) in OSCC patients. HR: hazard ratio estimated using Cox proportional hazards modeling. CI: 95% confidence interval

**Fig. 5:**
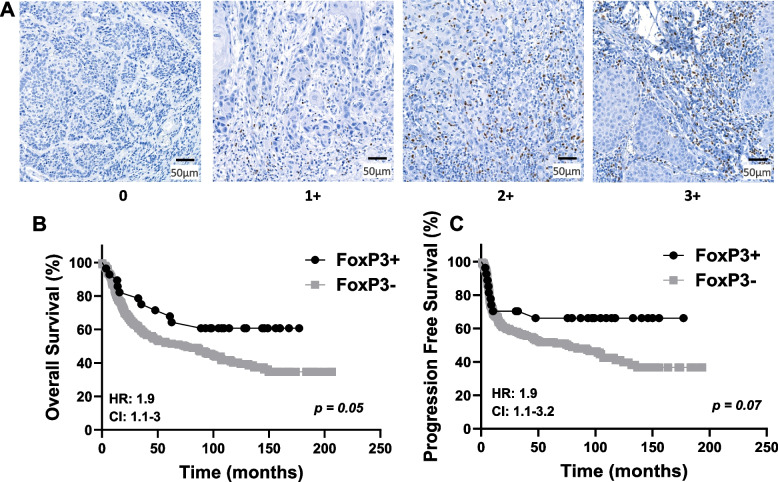
Prognostic Impact of FoxP3 Expression in OSCC. **A** Shown are images of negative [0], low [1 +], moderate [2 +], and strong [3 +] FoxP3 expression scores. **B**-**C** Shown are Kaplan–Meier estimates of Overall Survival (**B**) and Progression Free Survival (**C**) according to FoxP3 + (immunoscores of 2 + and 3 +) and FoxP3- (immunoscores of 0 and 1 +) in OSCC patients. HR: hazard ratio estimated using Cox proportional hazards modeling. CI: 95% confidence interval

The 5-year survival rate for all OSCC patients was 54% and the OS rate was 41% over an average follow up time of 139.6 months (11.6 years). Evaluation of OS and PFS based on TIL expression revealed that CD3 + (Fig. 2), CD4 + (Fig. 3), and CD8 + (Fig. 4**)** expression were all significantly associated with more favorable OS and PFS compared to CD3- (OS: *p* = 0.003, PFS: *p* = 0.03), CD4- (OS: *p* < 0.003, PFS: *p* = 0.003) and CD8- (OS: *p* = 0.04, PFS: *p* = 0.02) expression respectively using univariate analyses (Table 5). FOXP3 + expression showed a trend toward a significant association with more favorable OS but not PFS (OS: *p* = 0.05, PFS: *p* = 0.07) (Fig. 5**, **Table 5) using univariate analysis**.** However, the significant differences in TIL survival outcomes observed in the univariate analysis, was not observed in the multivariate analysis (Table 6). When survival outcomes were analyzed according to the patients’ clinicopathological characteristics, we found that higher T-stage (T2, T3, and T4) (OS: *p* < 0.001, PFS: *p* = 0.02), lymph node-positive (OS: *p* < 0.001, PFS: *p* < 0.001), poor tumor differentiation (OS: *p* < 0.001, PFS: *p* < 0.001), PNI (OS: *p* < 0.001, PFS: *p* < 0.001), and LVI (OS: *p* < 0.001, PFS: *p* = 0.008) were significantly associated with worse OS and PFS in the univariate analysis (Table 5). The presence of bone invasion was significantly associated with worse OS (but not PFS) in the univariate analysis (OS: *p* = 0.002, PFS: *p* = 0.31) (Table 5). On the other hand, in the multivariate analysis, only lymph node-positive (OS: *p* < 0.001, PFS: *p* < 0.001) and poor tumor differentiation (OS: *p* = 0.03, PFS: *p* = 0.04) remained significantly associated with OS and PFS; and PNI remained associated with PFS (*p* = 0.04) but not OS (*p* = 0.06) (Table 6). These results suggest that the TIL markers analyzed here are not independent predictors of survival outcomes; and conventional markers such as nodal status, tumor differentiation and presence of PNI emerged as significant independent predictors for survival outcomes in OSCC patients.

**Table 5:** Univariate analyses of prognostic factors for Overall Survival and Progression Free Survival

	**Overall Survival**				**Progression Free Survival**			
	**HR**	**95% CI**	*p*-value*	***p*-value**	**HR**	**95% CI**	*p*-value*	***p*-value**
Sex								
Male	1	0.6–1.2	0.31		1	0.7–1.5	0.91	
Female	0.8				1			
Location								
Tongue	1			0.16	1			0.5
FOM	1.6	0.9–2.9	0.13		0.5	1.6	0.61	
Gingiva	1.2	0.7–2.2	0.49		0.7	0.4–1.2	0.26	
Smoking status								
None	1			0.48	1			0.84
Quit	1	0.6–1.6	0.89		0.9	0.6–1.5	0.7	
Active	1.2	0.8–1.8	0.39		0.9	0.6–1.4	0.53	
T stage								
T1	1			< 0.001	1			0.02
T2	1.7	1–2.9	0.04		1.8	1.1–3.1	0.02	
T3/T4	2.6	1.7–3.8	< 0.001		1.8	1.2–2.8	0.008	
N stage								
No	1	2–4.2	< 0.001		1	1.8–4	< 0.001	
Yes	2.9				2.7			
Differentiation								
Well	1				1			
Mod	1.4	0.9–2.2	0.2	< 0.001	1.3	0.8–2.2	0.32	< 0.001
Poor	2.6	1.6–4.5	< 0.001		2.5	1.4–4.4	0.0013	
Perineural invasion								
No	1	1.4–2.8	< 0.001		1	1.5–3.2	< 0.0001	
Yes	2				2.2			
Lymphovascular invasion								
No	1	1.4–3	< 0.001		1	1.1–2.9	0.008	
Yes	2				1.8			
Bone invasion								
No	1	1.2–2.5	0.002		1	0.8–1.8	0.31	
Yes	1.7				1.2			
CD3								
High	1	1.2–2.4	0.003		1	1.1–2.3	0.03	
Low	1.7				1.6			
CD8								
High	1	1.1–2.4	0.04		1	1.2–2.7	0.02	
Low	1.6				1.8			
CD4								
High	1	1.5–2.9	< 0.001		1	1.2–2.5	0.003	
Low	2				1.8			
FOXP3								
High	1	1.1–3	0.05		1	1.1–3.2	0.07	
Low	1.9				1.9			

*HR* hazard ratio, *CI* 95% confidence interval, *p value compared to top row of respective factor

**Table 6:** Multivariate analyses of prognostic factors for Overall Survival and Progression Free Survival

	**Overall Survival**			**Progression Free Survival**		
	**HR**	**95% CI**	***p*-value**	**HR**	**95% CI**	***p*-value**
**T stage**						
T1	1	0.7–1.4	0.96	1	0.6–1.3	0.54
T2/T3/T4	1			0.9		
**N stage**						
No	1	1.4–2.7	< 0.001	1	1.3–2.6	< 0.001
Yes	2			1.8		
**Differentiation**						
Well-mod	1	1–1.9	0.07	1	1–2.1	0.0496
Poor	1.4			1.5		
**Perineural invasion**						
No	1	0.9–1.7	0.14	1	1.2–2.3	< 0.01
Yes	1.3			1.6		
**Lymphovascular invasion**						
No	1	0.8–1.5	0.56	1	0.7–1.5	0.81
Yes	1.1			1		
**CD3**						
High	1	0.7–1.6	0.75	1	0.7–1.6	0.84
Low	0.8			1		
**CD8**						
High	1	0.9–2.4	0.16	1	0.7–2.1	0.41
Low	1.5			1.2		
**CD4**						
High	1	0.8–1.8	0.39	1	0.7–1.6	0.76
Low	1			1.1		
**FOXP3**						
High	1	0.5–1.4	0.48	1	0.4–1.2	0.18
Low	1			0.7		

*HR* hazard ratio, *CI* 95% confidence interval

### Prognostic impact by TIL expression based on clinical stage

**Fig. 6:**
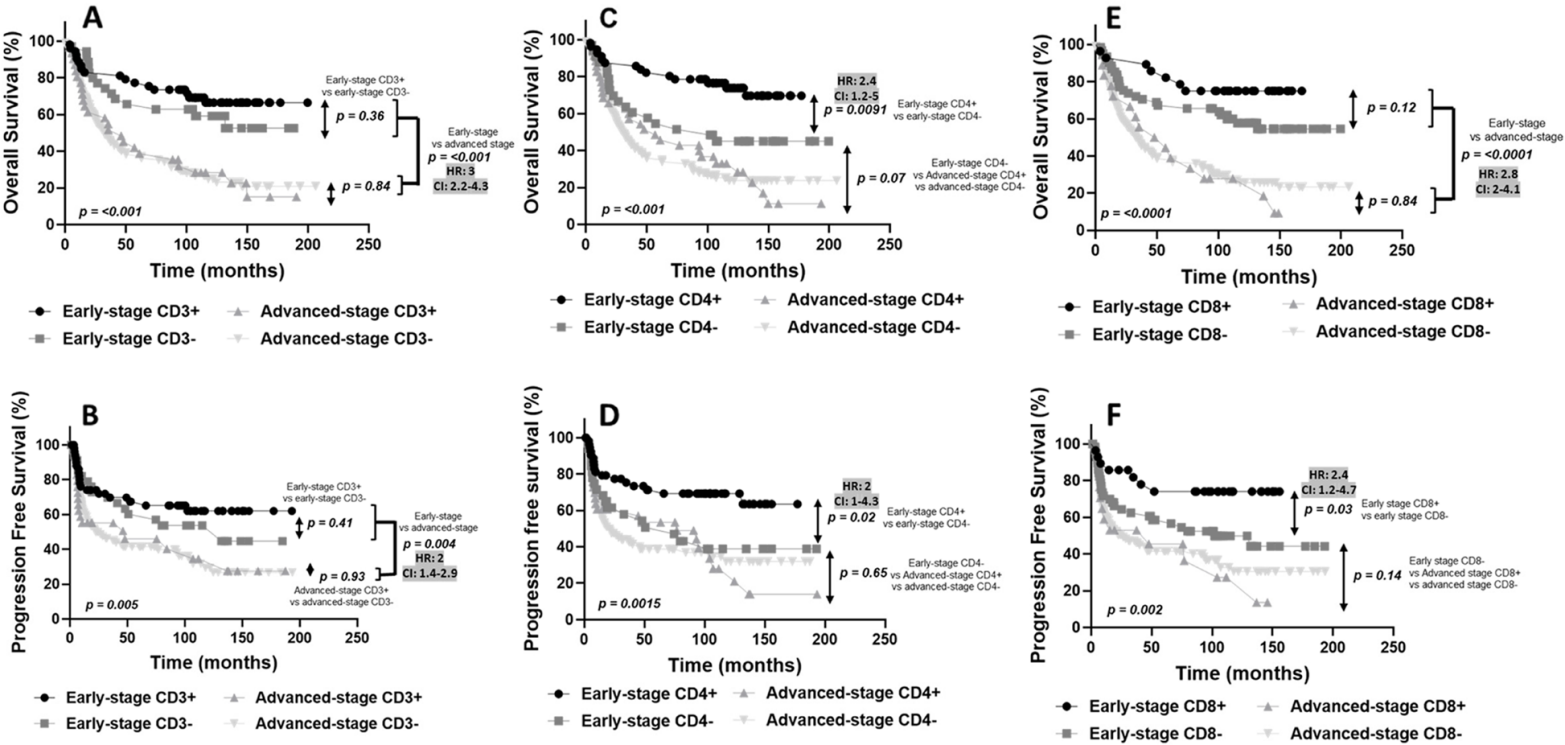
Prognostic Value of CD3, CD4 and CD8 Expression Based on Early and Advanced Stage. Shown are Kaplan–Meier estimates of Overall Survival (**A**,**C**,**E**) and Progression Free Survival (**B**,**D**,**F**) according to stage (Early or Advanced) and CD3 (**A**,**B**), CD4 (**C**,**D**), and CD8 (**E**,**F**) expression in OSCC patients. HR, hazard ratio; CI, 95% confidence interval

**Fig. 7:**
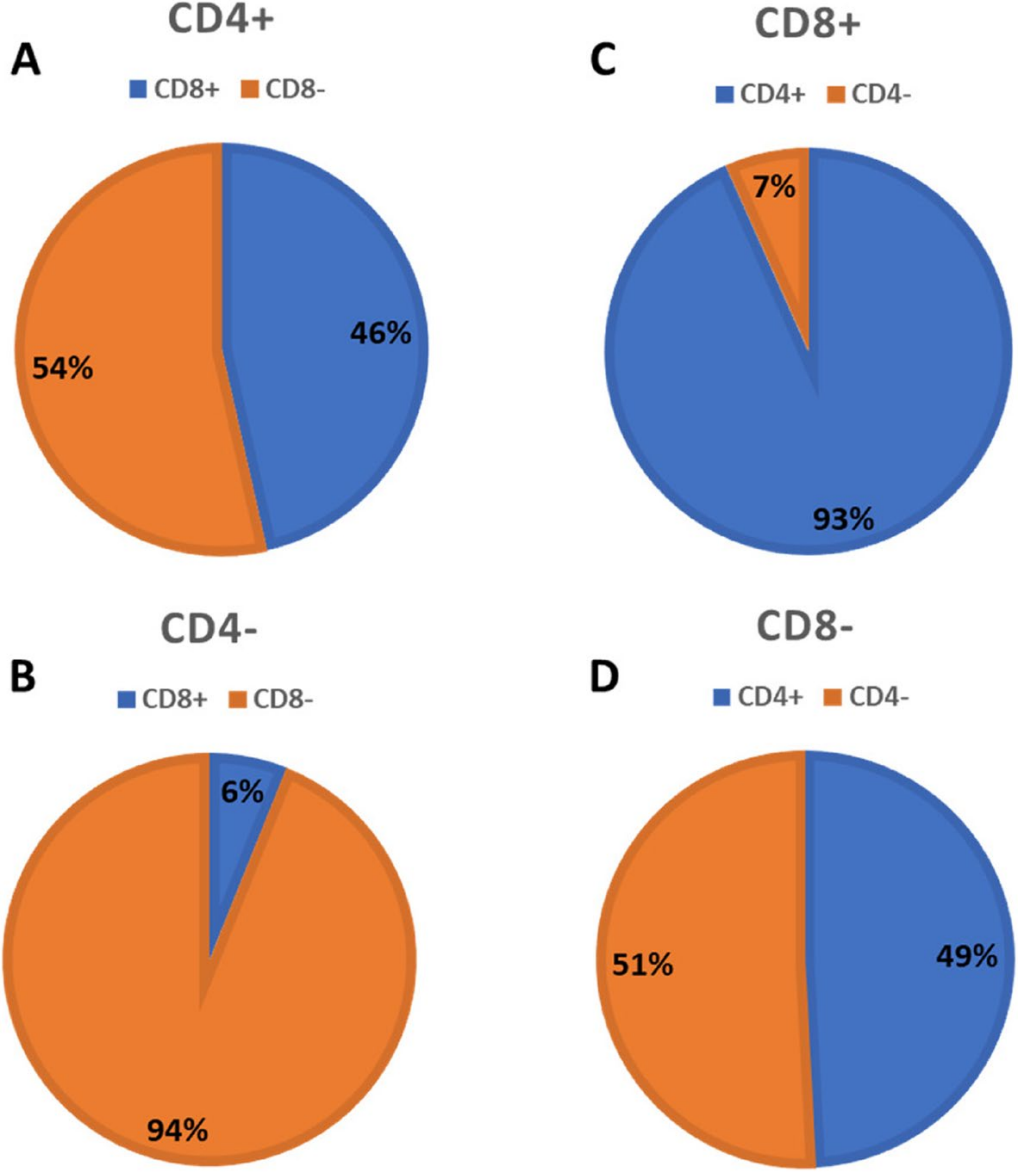
CD4 and CD8 Expression in Early Stage OSCCs. Shown are percentages of early stage CD4 + (**A**) and CD4- (**B**) OSCC tumors with CD8 + and CD8- expression; and percentages of early stage CD8 + (**C**) and CD8- (**D**) OSCC tumors with CD4 + and CD4- expression

It is well known that early diagnosis of OSCC can greatly improve upon patient prognosis. However, even for early-stage patients, the 5-year survival rate can be as low as 60% [29–31]. Therefore, predictive biomarkers remain necessary to stratify OSCC patients into high and low risk categories for recurrence. In the present cohort, the 5-year survival rate for early-stage patients was 72% over an average follow-up time of 133 months, compared to a 5-year survival rate of 40% for advanced-stage patients over an average follow-up time of 141 months. As expected, patients with advanced stage OSCC displayed significantly worse OS and PFS compared to early-stage patients (OS: *p* = < 0.0001, HR = 3, 95% CI 2.2–4.3; PFS: *p* = 0.0004, HR = 2, 95%CI 1.4–2.9) (SupplementalFig. 6); and was associated with moderate/poor differentiation (*p* < 0.001), PNI (*p* < 0.001), LVI (*p* < 0.001) and the presence of distant metastases (*p* = 0.04) (Table 7). We next separated early and advanced-stage patients by their TIL expression status. For CD3 expression we observed that early-stage OSCCs with CD3 + expression had significantly less PNI compared to the early-stage OSCCs with CD3- expression (*p* = 0.02, Table 7). However, there were no significant differences in OS between CD3 + and CD3- expression in early (*p* = 0.36) or advanced stage patients (*p* = 0.84) (Fig. 6A). A similar result for CD3 expression was observed for PFS in early (*p* = 0.41) and advanced stage (*p* = 0.93) patients (Fig. 6B). For CD4 expression, we found that OSCCs with moderate/poorly differentiated features were significantly more likely to be found in early-stage CD4- OSCCs compared to early-stage CD4 + OSCCs (*p* = 0.03, Table 8). Additionally, early-stage OSCC patients with CD4- expression had significantly worse OS (*p* = < 0.0091, HR = 2.4, 95% CI 1.2–5) (Fig. 6C) and PFS (*p* = 0.02, HR = 2.1, 95% CI 1–4.3) (Fig. 6D) compared to early-stage OSCC patients with CD4 + expression. Interestingly, the OS and PFS for the early-stage CD4- OSCC patients were similar to that of advanced-stage CD4 + and CD4- patients (OS: *p* = 0.07, PFS: *p* = 0.65) (Fig. 6C,D) suggesting that low or lack of CD4 expression may select for early-stage patients with poor prognosis. For CD8 expression there was a significant difference in the age at diagnosis among the advanced stage OSCC patients, where CD8 + patients were older at diagnosis than CD8- patients (*p* = 0.04, Table 9). Otherwise, there were no other significant differences observed in clinicopathological outcomes among the early and advanced stages based on CD8 expression. Similar to CD3, there were no significant differences in OS between CD8 + and CD8- expression in early (*p* = 0.12) or advanced stage patients (*p* = 0.84) (Fig. 6E). However early stage CD8- patients had significantly worse PFS than early stage CD8 + patients (*p* = < 0.03, HR = 2.4, 95% CI 1.2–4.7) and was similar to that of advanced-stage CD8 + and CD8- patients (*p* = 0.14, Fig. 6F). FOXP3 was not evaluated based on clinical stage due to the low number of OSCC cases that were FOXP3 + . Given that CD4 expression appeared to be the best predictor of both OS and PFS in early stage OSCC patients (Fig. 6C,D), we next asked the question of whether CD4 expression coincided with CD8 expression in early stage tumors. In the early-stage CD4 + tumors, there was an almost equal distribution of CD8 + (46%) and CD8- (54%) expression (Fig. 7A). In the early-stage CD4- tumors, the majority of these tumors were also CD8- (94%) with only 6% being CD8 + **(**Fig. 7B**).** Likewise in the early-stage CD8 + tumors, the majority (93%) of cases were also CD4 + (Fig. 7C) but in the early-stage CD8- tumors, there was an almost equal distribution of CD4 + (49%) and CD4- cases (51%) (Fig. 7D).

**Table 7:** Clinicopathological Characteristics of Early and Advanced OSCC Stages Based on CD3 Expression

	**Early Stage**			**Advanced Stage**		
	**CD3 + ** **(*n* = 53)**	**CD3-** **(*n*** **= 35)**	***p*-value**	**CD3 + ** **(*n*** **= 31)**	**CD3-** **(*n*-102)**	***p*-value**
Sex						
Male	28 (52.8%)	18 (51.4%)	0.88	19 (61.3%)	65 (63.7%)	0.81
Female	25 (47.2%)	17 (48.6%)		12 (38.7%)	37 (36.3%)	
	*p*-value = 0.45*					
Age						
Avg [± stdev]**	59.8 [15.5]	64.5 [12.3]	0.12	64 [15.8]	62.3 [12.6]	0.59
	*p*-value = 0.37*					
Smoking history						
Active	23 (44.2%)	16 (45.7%)	0.8	18 (58.1%)	53 (52%)	0.62
Never	15 (28.9%)	8 (22.9%)		5 (16.1%)	25 (24.5%)	
Former	14 (26.9%)	11 (31.4%)		8 (25.8%)	24 (23.5%)	
	*p*-value = 0.82*					
Location^#^						
Tongue	35 (77.8%)	15 (60%)	0.11	11 (57.9%)	25 (44.6%)	0.61
Floor of mouth	4 (8.9%)	7 (28%)		4 (21.1%)	16 (28.6%)	
Gingiva	6 (13.3%)	3 (12%)		4 (21.1%)	15 (26.8%)	
	*p*-value = 0.04*					
Differentiation						
Well	19 (37.3%)	7 (20%)	0.07	0 (0%)	9 (8.9%)	0.12
Moderate	28 (54.9%)	20 (57.1%)		25 (80.7%)	65 (64.4%)	
Poorly	4 (7.9%)	8 (22.9%)		6 (19.4%)	27 (26.7%)	
	*p*-value = < 0.001*					
Perineural invasion						
Yes	10 (20.8%)	15 (44.1%)	0.02	17 (56.7%)	56 (56.6%)	0.99
No	38 (79.2%)	19 (55.9%)		13 (43.3%)	43 (43.4%)	
	*p*-value = < 0.001*					
Lymphovascular invasion						
Yes	6 (12.2%)	4 (11.8%)	0.95	11 (37.9%)	48 (48%)	0.34
No	43 (87.8%)	30 (88.2%)		18 (62.1%)	52 (52%)	
	*p*-value = < 0.001*					
Local recurrence						
Yes	9 (22%)	8 (30.8%)	0.42	10 (45.5%)	21 (29.2%)	0.15
No	32 (78%)	18 (69.2%)		12 (54.5%)	51 (70.8%)	
	*p*-value = 0.28*					
Distance metastasis						
Yes	4 (10.3%)	2 (8%)	0.76	5 (22.7%)	22 (29.3%)	0.54
No	35(89.7%)	23 (92%)		17 (77.3%)	53 (70.7%)	
	*p*-value = 0.04*					

^*^early vs advanced stage

^**^Average age at diagnosis

^#^ OSCC on overlapping/other locations were not included in Chi-square test

**Table 8:** Clinicopathological Characteristics of Early and Advanced OSCC Stages Based on CD4 Expression

	**Early Stage**			**Advanced Stage**		
	**CD4 + ** **(*n*** **= 56)**	**CD4-** **(*n*** **= 33)**	***p*-value**	**CD4 + ** **(*n*** **= 35)**	**CD4-** **(*n*** **= 100)**	***p*-value**
Sex						
Male	28 (50%)	19 (57.6%)	0.49	22 (62.9%)	64 (64%)	0.9
Female	28 (50%)	14 (42.4%)		13 (37.1%)	36 36%)	
	*p*-value = 0.37*					
Age						
Avg [± stdev]**	60.3 [14.2]	63.1 [15.2]	0.4	67.1 [13.4]	61.5 [12.8]	0.04
	*p*-value = 0.12*					
Smoking history						
Active	24 (43.6%)	16 (48.5%)	0.9	19 (54.3%)	52 (52%)	0.93
Never	15 (27.3%)	8 (24.2%)		7 (20%)	23 (23%)	
Former	16 (29.1%)	9 (27.3%)		9 (25.7%)	25 (25%)	
	*p*-value = 0.96*					
Location^#^						
Tongue	28 (73.7%)	13 (52%)	0.14	8 (42.1%)	30 (51.7%)	0.11
Floor of mouth	4 (10.5%)	7 (28%)		3 (15.8%)	17 (29.3%)	
Gingiva	6 (15.8%)	5 (20%)		8 (42.1%)	11 (19%)	
	*p*-value = 0.06*					
Differentiation						
Well	19 (35.2%)	7 (21.2%)	0.03	1 (2.9%)	8 (8.1%)	0.45
Moderate	31 (57.4%)	17 (51.5%)		26 (74.3%)	64 (64.7%)	
Poorly	4 (7.4%)	9 (27.3%)		8 (22.9%)	27 (27.3%)	
	*p*-value = < 0.001*					
Perineural invasion						
Yes	13 (26%)	12 (36.4%)	0.31	14 (41.2%)	58 (59.8%)	< 0.001
No	37 (74%)	21 (63.6%)		20 (58.8%)	39 (40.2%)	
	*p*-value = < 0.001*					
Lymphovascular invasion						
Yes	5 (9.8%)	5 (15.2%)	0.46	9 (26.5%)	50 (51.5%)	0.01
No	46 (90.2%)	28 (84.8%)		25 (73.5%)	47 (48.5%)	
	*p*-value = < 0.001*					
Local recurrence						
Yes	9 (21.4%)	9 (34.6%)	0.23	9 (36%)	23 (33.3%)	0.81
No	33 (78.6%)	17 (65.4%)		16 (64%)	46 (66.7%)	
	*p*-value = 0.49*					
Distance metastasis						
Yes	4 (10%)	2 (8%)	0.79	5 (19.3%)	22 (31%)	0.25
No	36 (90%)	23 (92%)		21 (80.8%)	49 (69%)	
	*p*-value = 0.02*					

^*^early vs advanced stage

^**^Average age at diagnosis

^#^ OSCC on overlapping/other locations were not included in Chi-square test

**Table 9:** Clinicopathological Characteristics of Early and Advanced OSCC Stages Based on CD8 Expression

	**Early Stage**			**Advanced Stage**		
	**CD8 + ** **(*n*** **= 28)**	**CD8-** **(*n*** **= 61)**	***p*-value**	**CD8 + ** **(*n*** **= 18)**	**CD8-** **(*n*** **= 114)**	***p*-value**
Sex						
Male	12 (42.9%)	35 (57.4%)	0.2	10 (55.6%)	73 (64%)	0.49
Female	16 (57.1%)	26 (42.6%)		8 (44.4%)	41 (36%)	
	*p-value* = 0.23*					
Age						
Avg [± stdev]**	63.4 [13.6]	60.4 [15]	0.37	69.2 [13.5]	61.9 [13.2]	0.04
	*p-value* = 0.12*					
Smoking history						
Active	11 (39.3%)	29 (48.3%)	0.15	8 (44.4%)	61 (53.5%)	0.39
Never	11 (39.3%)	12 (20%)		3 (16.7%)	26 (22.8%)	
Former	6 (21.4%)	19 (31.7%)		7 (38.9%)	27 (23.7%)	
	*p*-value = 0.33*					
**Location**^**#**^						
Tongue	17 (81%)	34 (68%)	0.27	5 (62.5%)	33 (48.5%)	0.66
Floor of mouth	1 (4.8%)	10 (20%)		1 (12.5%)	18 (26.5%)	
Gingiva	3 (14.3%)	6 (12%)		2 (25%)	17 (25%)	
	*p*-value = 0.11*					
Differentiation						
Well	11 (42.3%)	15 (24.6%)	0.08	0 (0%)	9 (8%)	0.39
Moderate	14 (53.9%)	34 (55.7%)		14 (77.8%)	74 (65.5%)	
Poorly	1 (3.9%)	12 (19.7%)		4 (22.2%)	30 (26.5%)	
	*p* = < 0.001*					
Perineural invasion						
Yes	4 (16.7%)	21 (35.6%)	0.09	9 (50%)	64 (58.2%)	0.52
No	20 (83.3%)	38 (64.4%)		9 (50%)	46 (41.8%)	
	*p* = < 0.001*					
Lymphovascular invasion						
Yes	1 (4%)	9 (15.3%)	0.15	8 (47.1%)	51 (46%)	0.93
No	24 (96%)	50 (84.7%)		9 (52.9%)	60 (54.1%)	
	*p* = < 0.001*					
Local recurrence						
Yes	4 (16%)	14 (32.6%)	0.14	5 (38.5%)	26 (32.5%)	0.67
No	21 (84%)	29 (67.4%)		8 (61.5%)	54 (67.5%)	
	*p* = 0.38*					
Distance metastasis						
Yes	2 (8.3%)	4 (9.8%)	0.85	5 (35.7%)	21 (25.6%)	0.43
No	22 (91.7%)	37 (90.2%)		9 (64.3%)	61 (74.4%)	
	*p* = 0.04*					

^*^early vs advanced stage

^**^Average age at diagnosis

^**#**^OSCC on overlapping/other locations were not included in Chi-square test

## Discussion

Overall, the results presented here indicate that tumor differentiation and nodal status are independent prognostic indicators of survival outcomes in OSCC patients which align well with prior reports [32–35]. Unfortunately, in some cases, patients with identical tumor staging and/or clinicopathological characteristics can have vastly different survival outcomes and response to treatment. Additionally, conventional indicators like tumor differentiation and nodal status do not affect therapeutic decisions. The only predictive indicators in routine clinical use for OSCC are PD-L1 status and the combined positive score (CPS) for pembrolizumab administration. Therefore, the use of conventional indicators for risk stratification could benefit from additional biomarkers for treatment planning purposes and overall disease management.

Immune cells present in the tumor microenvironment (TME) can have either pro- or anti-tumor activity and their potential as biomarkers represent a promising avenue for investigation. Prior studies with TILs have shown that high expression of CD3 was associated with favorable clinicopathological characteristics and predictive of favorable survival outcomes in OSCC cases [25, 36–38]. These results align with our results showing that high CD3 expression was associated with favorable patient clinicopathological characteristics such as well-differentiated tumors, lower T-stage, absence of lymph node metastasis, absence of LVI, and early-stage disease (Table 3); and favorable survival outcomes (Fig. 2).

T cell subsets such as T-helper cells (CD4 +), cytotoxic T-cells (CD8 +), and regulatory T-cells (Tregs (FOXP3 +)) have also been explored as biomarkers for OSCCs. CD8 + T cells are generally considered as the main force against cancer. In support of this, Sale de Sa et al. found an association between high CD3 and CD8 expression with well-differentiated tumors [37], Mukherjee et al. found that early T-stage was associated with high CD3 expression, and Ahn et al. found that early T-stage was associated with high CD3 and CD8 expression [25, 36]. Mukherjee et al. also reported that high CD3 and high CD8 expression were associated with the absence of lymph node metastasis, which is consistent with the study of Cho et al. which found that low peritumoral CD8 expression was associated with lymph node metastasis [25, 39]. Based on these data, our results showing that high CD8 expression was associated with favorable patient characteristics (Table 3) and survival outcomes (Fig. 4) was expected and concur with previous findings in OSCC [24, 25, 37, 40–44].

For CD4 + T cells, prior studies have found an association between low CD4 expression and higher T stage and presence of LVI [39, 45] which agrees with our results (Table 3). However, 2 studies have reported findings that contradict our findings. Spector et al. and Gaafar et al., reported that higher CD4 + cell counts were associated with advanced-stage OSCC [41, 46]. Additionally, the study by Gaafar et al. found that dense CD4 + cell infiltrate was associated with poorly differentiated tumors. Regarding the prognostic value of CD4 expression in OSCCs, we show significant associations between high CD4 expression and favorable survival outcomes (Fig. 3). However, our results do not align with previous published findings. For example, Sales de Sa et al. found that increased CD4 + expression tended to have a worse prognosis in OSCC, but this result was not significant and the study only included 48 participants [37]. Gaafar et al. found that dense CD4 + cells were associated with worse OS, although it is important to note that the median follow-up time for the study was relatively short (48 months), and the sample size was relatively small (*n* = 22) [46]. At this time, we are unclear about these inconsistent CD4 expression results in OSCCs, however CD4 + cells are able to differentiate into different subsets such as Th1, Th2, Th9, Th17, Th22, Tregs, and Tfh (follicular helper T cells) which all have different roles. We speculate that differences in the tumor subtypes of CD4 + cells may explain the variability observed among these studies [43, 47], and suggests that analyzing the prognostic value of specific CD4 + T cell subsets may be of value.

We also investigated CD4 + Tregs due to their ability to impede effective immunity against cancers. CD4 + Tregs are characterized by the expression of the FOXP3 transcription factor; and studies by Liang et al. found that high FOXP3 expression was associated with T3-T4 tumors in OSCC occurring on the tongue. Gaafar et al. in also found that high intratumoral FOXP3 expression was associated with advanced stage and poorly differentiated tumors [48, 49]. Based on these prior data, it is no surprise that high FOXP3 expression has been associated with a worse survival rate [25–27, 36, 39–43, 45, 48, 50, 51]. However, like CD4 expression, previous published studies have reported conflicting findings. For example, Wolf et al. reported that high FOXP3 expression was associated with early T-stage, and Ahn et al. reported that low FOXP3 expression was associated with the presence of PNI, high T-stage, and overall stage [36, 50]. Our results tend to align with these aforementioned studies in that FOXP3 + OSCCs were associated with lower T-stage, absence of lymph node metastasis, and early-stage disease (Table 3) and thus a trend toward more favorable survival outcomes (*p* = 0.05, Fig. 5). The better outcomes observed in our study could be explained by two proposed concepts: 1: FOXP3 expression may not accurately represent the infiltration of Tregs, as not all Tregs express FOXP3 and not all FOXP3 + cells are Tregs and may have little or no suppressive function [52]; and 2: the infiltration of Tregs may play a role in preventing tumor invasion and metastasis by inhibiting harmful inflammatory processes that benefit tumor growth [26, 47, 52]. In fact, Koike et al. found that recurrence-free survival was favorable in OSCC patients with high FOXP3^+^ expression in the parenchyma of the invasive front and that FOXP3^+^ T cells may exert some type of site-specific anti-tumor effects [53]. Clearly further investigation is warranted before FOXP3 can be pursued as a prognostic biomarker for OSCC.

Although our results showing that high TIL marker (CD3, CD4 and CD8) expression were all significantly associated with favorable survival outcomes using univariate analysis (Table 5), none of these markers remained significant in the multivariate analysis (Table 6) suggesting these markers cannot independently predict OSCC patient outcomes. However, there are some studies in the OSCC literature that report that high CD8 levels were significantly associated with better outcomes using multivariate analysis [37, 42, 43]. Similarly, Zhou et al.reported that high levels of CD3 at the invasive margin and CD8 at the tumor center could independently predict better prognosis for patients [24]. Although most studies have reported no prognostic significance of CD4 in OSCC, one study by Nguyen et al. found that high levels of CD4 were independent predictors for improved OS and DSS in HNSCC [54]. Markedly contrary to our findings, Liang et al. found that high levels of FOXP3 were associated with worse survival in multivariate analysis [48]. Altogether, our work confirms the use of conventional factors such as tumor differentiation and nodal status as independent prognostic indicators of survival outcomes in OSCC patients. Given the variability in the results mentioned in the aforementioned studies with TIL marker expression, there remains the possibility that TIL expression may alternatively be used as prognostic indicators in combination with conventional factors to enhance risk stratification in OSCC patients.

As mentioned before, patients with identical tumor staging and/or clinicopathological characteristics can have vastly different survival outcomes and this is often observed in early-stage cases where 30–35% of early-stage OSCC patients can experience locoregional failure [9]. In fact, the 5-year survival rate for early-stage patients in our study was 72%, meaning despite having early-stage disease which is associated with favorable survival outcomes (SupplementalFig. 6), 28% of the patients were dead within 5 years. Therefore early-stage OSCC cases that exhibit recurrence and progression after surgical resection may have other characteristics that contribute to poor survival. One factor that can lead to poor prognosis in early-stage disease is undetected lymph node metastasis, which is a major prognostic indicator. Schilling et al. suggested that sentinel node biopsy may be a reliable method to detect lymph node metastasis in early-stage OSCC pateints [55]. However, currently not all OSCC patients undergo neck dissection which makes the possibility of misclassifying advanced stage cancer as early-stage cancer a concern [56]. We therefore conducted further analysis to assess the prognostic impact of TILs according to stage. We surprisingly found that low expression of CD4 (and not CD3 or CD8) could identify early-stage OSCC patients with unfavorable survival outcomes (OS and PFS) (Fig. 6). The survival of these early-stage CD4- patients was strikingly similar to that of the advanced CD4 + and CD4- OSCC patients. We are unclear as to why low CD4 expression in particular was associated with increased risk of undesired survival outcomes in early-stage patients but we did observe that this cohort had significantly more tumors with poor differentiation compared to early-stage CD4 + tumors (*p* = 0.03). It is possible that higher number of tumors with poor differentiation might contribute to the poor survival outcomes, as tumor differentiation was associated with worse PFS in the multivariate analysis (Table 6**, **SupplementalFig. 5A). Furthermore, almost all (94%) early stage CD4- cases were also CD8-. This result is potentially due to the fact that CD4 + T-helper cells induce the proliferation of CD8 + cytotoxic T-cells, therefore when there is a low number of CD4 + T cells, the number of CD8 + T cells is also low [21]. As such, the poor survival of patients in this group might also be from lack of host immunity. Further robust validation is necessary to confirm the prognostic role of CD4 expression in early stage OSCC patients.

Other interesting findings from this data were that early-stage and lower T-stage disease (T1/T2) is more commonly observed on the tongue compared to the other disease sites (Table 2). These findings are consistent with a study by Suresh et al., which found that OSCC at the tongue tended to be in the early T Stage [57]. It is possible that due to the tongue being an easily visible, accessible and sensitive location, tumors and other abnormalities on the tongue can be found early (i.e. when at a small size) by self-exam, or during routine doctor/dentist exams. Despite the tongue being significantly associated with early-stage OSCC, we found no difference in survival outcomes between patients with tongue, FOM and gingiva (Table 5**, **SupplementalFig. 4).

We found that the FOM location was significantly associated with advanced (T3/T4) tumors; but also associated with male gender and active smoking (Table 2). These findings support previous studies reporting that tobacco smoking together with alcohol consumption was associated with OSCC at the FOM [58, 59]. The FOM likely has more exposure to carcinogens from tobacco compared to other OSCC sites due to the pool of saliva accumulating in this anatomical location. Mucosal thickness and local irritation may also be factors that are involved and are worth further study [60]. Lastly, we found that the FOM OSCCs were significantly associated with low expression of all the TIL markers tested (CD3, CD4, CD8, and FOXP3 (Table 4)). Given that regular tobacco use has been associated with an immunosuppressive TME [42, 61, 62]. We hypothesize that the constant exposure to tobacco may result in immunosuppression at the FOM location resulting in decreased T cell-mediated anti-tumor immunity. Further research is needed to explore the relationship between active smoking and the incidence of floor of mouth cancer, as well as the association between active smoking and TILs.

Our studies contain some limitations including:

- TILs were evaluated using TMAs, which may not accurately reflect the distribution or density of TILs in the entire tissue slide. Nevertheless, the use of TMAs allows for rapid evaluation of a large cohort and enables multiple tissue cores from different cases to undergo analysis or staining procedures simultaneously [63].
- The location of TILs was not considered in this analysis. Different immune cell types may be located in the invading edge and periphery of the tumor compared to the center of the tumor [24, 44], or in the stroma [40]. Therefore, analysis based on the location of immune cells may add to the prognostic value of TIL markers.
- By using CD3 to detect pan-T-cells, we cannot observe the subtypes of T-cells in the same tissue slide. Further investigation using double immunohistochemistry staining and multiplex immunofluorescence might be warranted. These techniques would allow investigators to observe the positivity of cells from more than one marker [26, 61]. The ratio of cytotoxic T-cells to T-helper cells or Treg cells might provide interesting information regarding patient outcome.
- CD4 + T cells consist of multiple subpopulations that have anti-tumor functions or suppressive functions [21, 64]. In this study, we only used FOXP3 to identify Tregs, which ignores the possible contribution of other types of CD4 + T cells.
- Our studies have not assessed other immune cells (including B cells) that play important roles in the immune response to cancer [12, 65]. For example, there is evidence of an antigen-driven immune response within the tumor caused by B-cells [66]. Further studies with a more comprehensive panel of immune markers are needed to better understand the role of immune cells in OSCC survival outcomes.
- Our study does not account for the functional activity of TILs, such as their ability to produce cytokines or lyse tumor cells. Additionally, we have no information regarding the genetic alterations present in the OSCC tumors used in this study. It is possible that connecting select TIL markers to specific gene expression profiles or functional genetic networks [67], may offer a more robust prognostic information for OSCC patients.
- All the cases in this study are from a single institution, which may limit the generalizability of our findings.

## Conclusions

Overall, our results confirm that conventional indicators such as lymph node metastasis, tumor differentiation, and PNI remain strong prognostic markers for OSCC patients. Evaluating TIL markers such as CD3, CD4, and CD8 can be useful in predicting patient survival, but should be used carefully in combination with the above conventional indicators given their lack of independent prognostic value. Finally, the expression of CD4 in particular may be helpful to identify early-stage OSCC patients with high or low risk of cancer recurrence and/or progression. Therefore, with additional validation, CD4 expression may influence treatment planning and decision making for early-stage OSCC patients.

### Supplementary Information

Supplemantary material 1.

Supplemantary material 2.

Supplemantary material 3.

Supplemantary material 4.

Supplemantary material 5.

Supplemantary material 6.

## Data Availability

The raw data supporting the conclusions of this article will be made available by the authors, upon reasonable request.
