# Supplementary material for: Prognostic Role of Tumor-Infiltrating Lymphocytes in Oral Squamous Cell Carcinoma
Source: BMC Cancer. 2024 Jun 26;24:766. doi: 10.1186/s12885-024-12539-5 (PMC11201865; doi:10.1186/s12885-024-12539-5)
Supplement: Supplementary file 2 — Supplemantary material 2. [file 12885_2024_12539_MOESM2_ESM.pptx]

## Slide 1
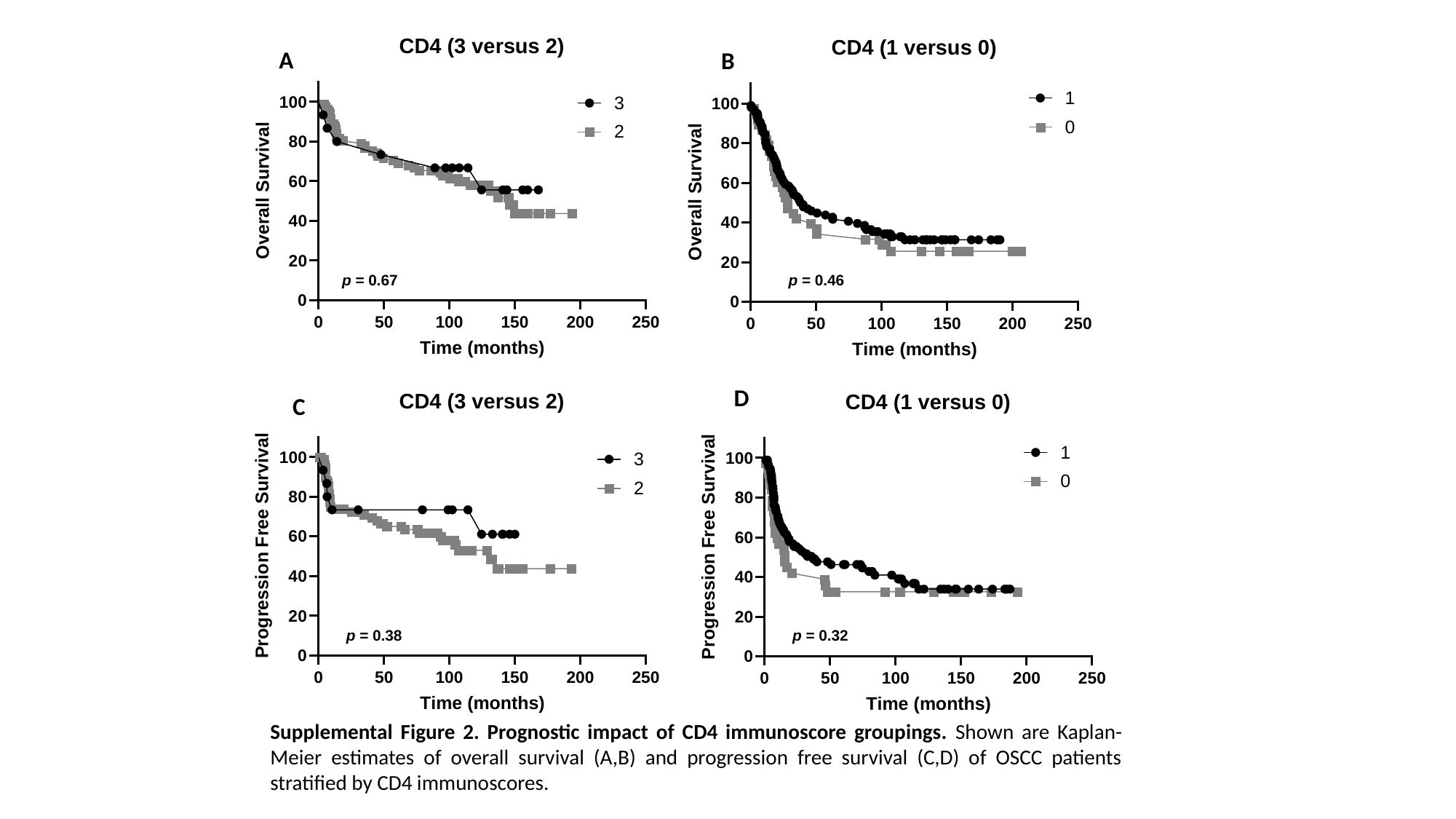

A
B
| p = 0.67 |
| --- |
| p = 0.46 |
| --- |
D
C
| p = 0.38 |
| --- |
| p = 0.32 |
| --- |
Supplemental Figure 2. Prognostic impact of CD4 immunoscore groupings. Shown are Kaplan-Meier estimates of overall survival (A,B) and progression free survival (C,D) of OSCC patients stratified by CD4 immunoscores.
