# Supplementary material for: Prognostic Role of Tumor-Infiltrating Lymphocytes in Oral Squamous Cell Carcinoma
Source: BMC Cancer. 2024 Jun 26;24:766. doi: 10.1186/s12885-024-12539-5 (PMC11201865; doi:10.1186/s12885-024-12539-5)
Supplement: Supplementary file 4 — Supplemantary material 4. [file 12885_2024_12539_MOESM4_ESM.pptx]

## Slide 1
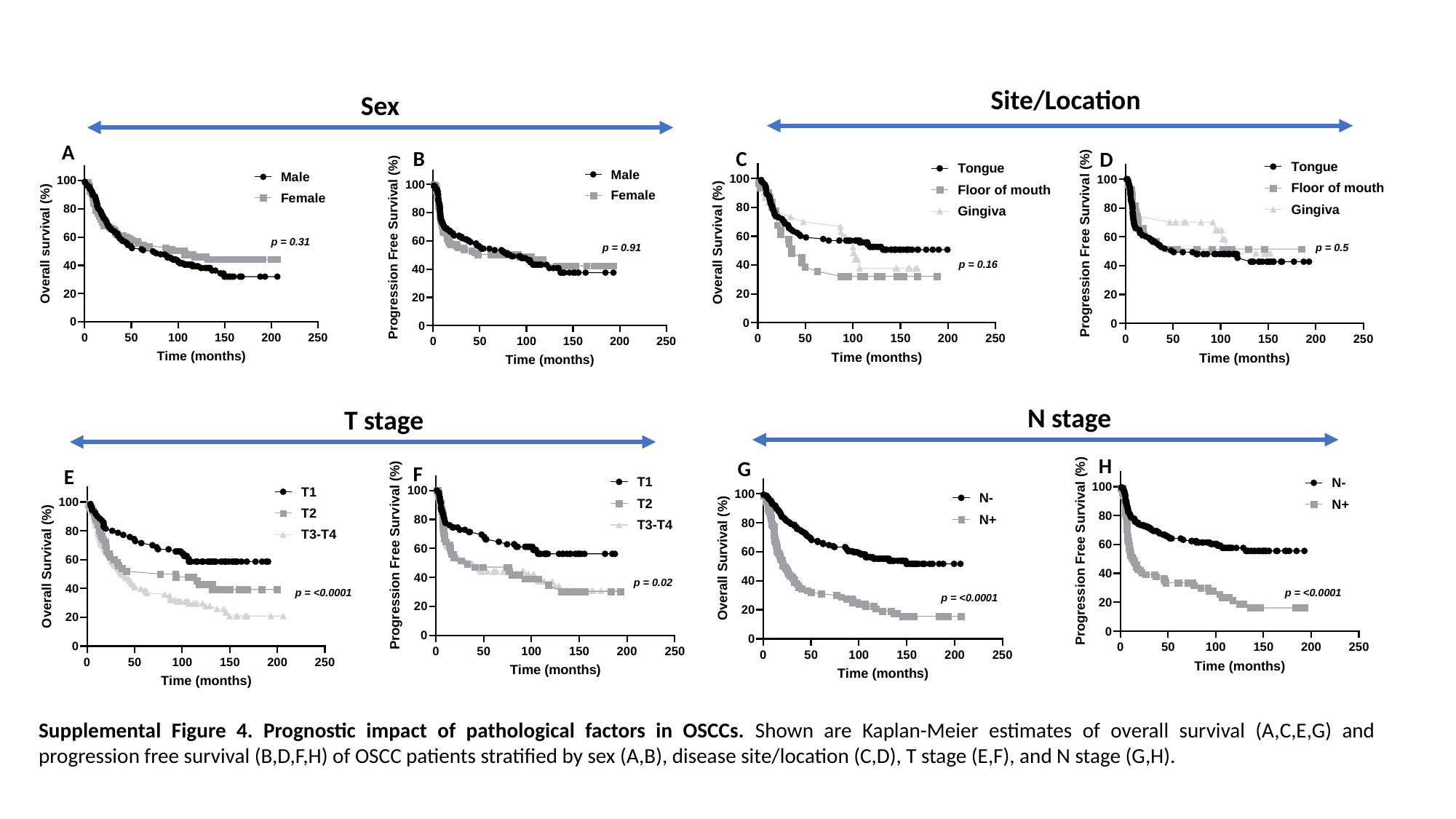

Site/Location
Sex
A
C
B
D
p = 0.31
p = 0.91
p = 0.5
p = 0.16
N stage
T stage
p = <0.0001
H
G
F
p = <0.0001
E
p = 0.02
p = <0.0001
Supplemental Figure 4. Prognostic impact of pathological factors in OSCCs. Shown are Kaplan-Meier estimates of overall survival (A,C,E,G) and progression free survival (B,D,F,H) of OSCC patients stratified by sex (A,B), disease site/location (C,D), T stage (E,F), and N stage (G,H).
