# Supplementary material for: Prognostic Role of Tumor-Infiltrating Lymphocytes in Oral Squamous Cell Carcinoma
Source: BMC Cancer. 2024 Jun 26;24:766. doi: 10.1186/s12885-024-12539-5 (PMC11201865; doi:10.1186/s12885-024-12539-5)
Supplement: Supplementary file 5 — Supplemantary material 5. [file 12885_2024_12539_MOESM5_ESM.pptx]

## Slide 1
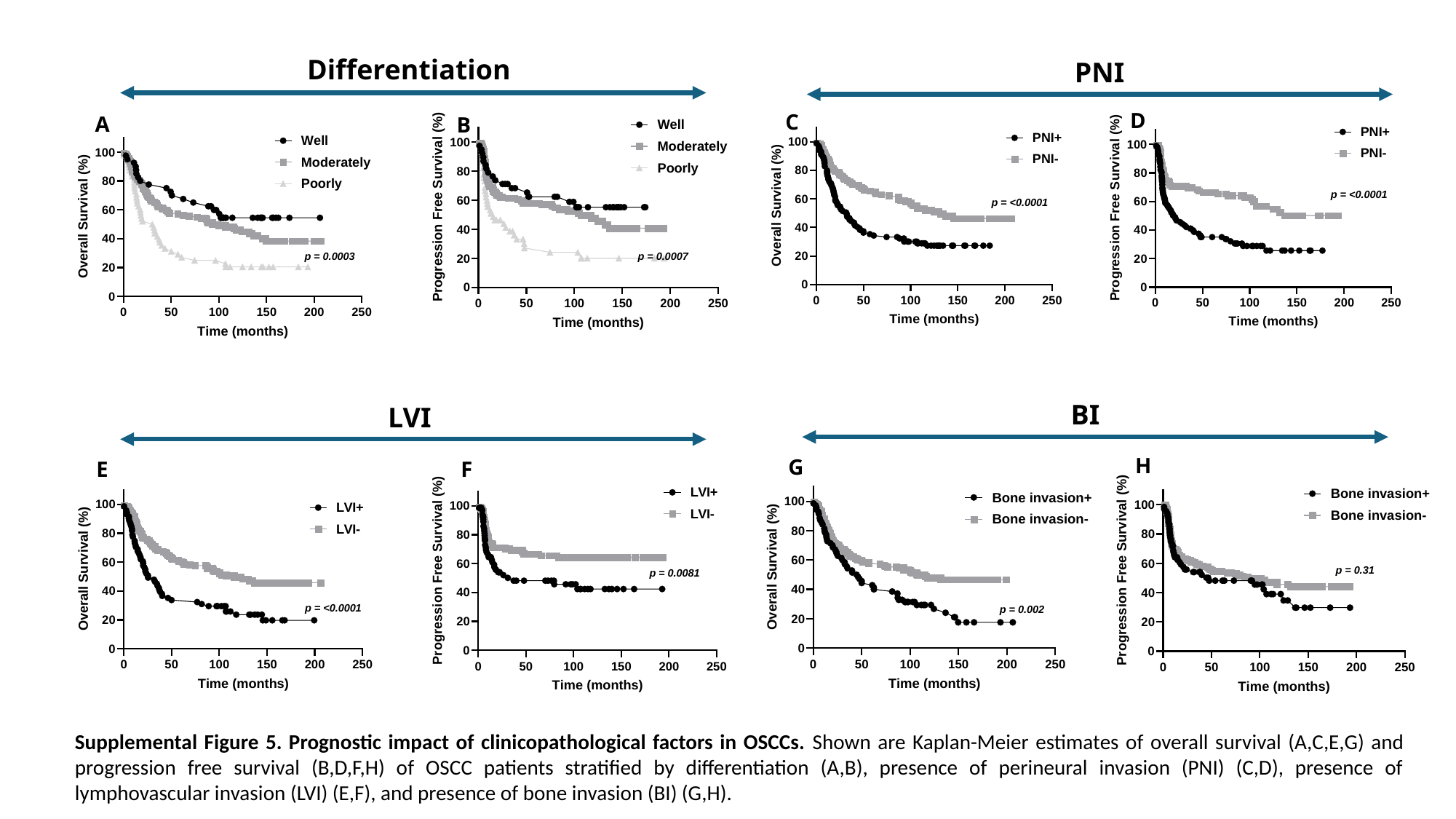

Differentiation
PNI
p = 0.0007
D
C
p = <0.0001
p = <0.0001
A
B
p = 0.0003
BI
LVI
H
G
F
E
p = 0.31
p = 0.002
p = 0.0081
p = <0.0001
Supplemental Figure 5. Prognostic impact of clinicopathological factors in OSCCs. Shown are Kaplan-Meier estimates of overall survival (A,C,E,G) and progression free survival (B,D,F,H) of OSCC patients stratified by differentiation (A,B), presence of perineural invasion (PNI) (C,D), presence of lymphovascular invasion (LVI) (E,F), and presence of bone invasion (BI) (G,H).
