# Supplementary material for: Prognostic Role of Tumor-Infiltrating Lymphocytes in Oral Squamous Cell Carcinoma
Source: BMC Cancer. 2024 Jun 26;24:766. doi: 10.1186/s12885-024-12539-5 (PMC11201865; doi:10.1186/s12885-024-12539-5)
Supplement: Supplementary file 6 — Supplemantary material 6. [file 12885_2024_12539_MOESM6_ESM.pptx]

## Slide 1
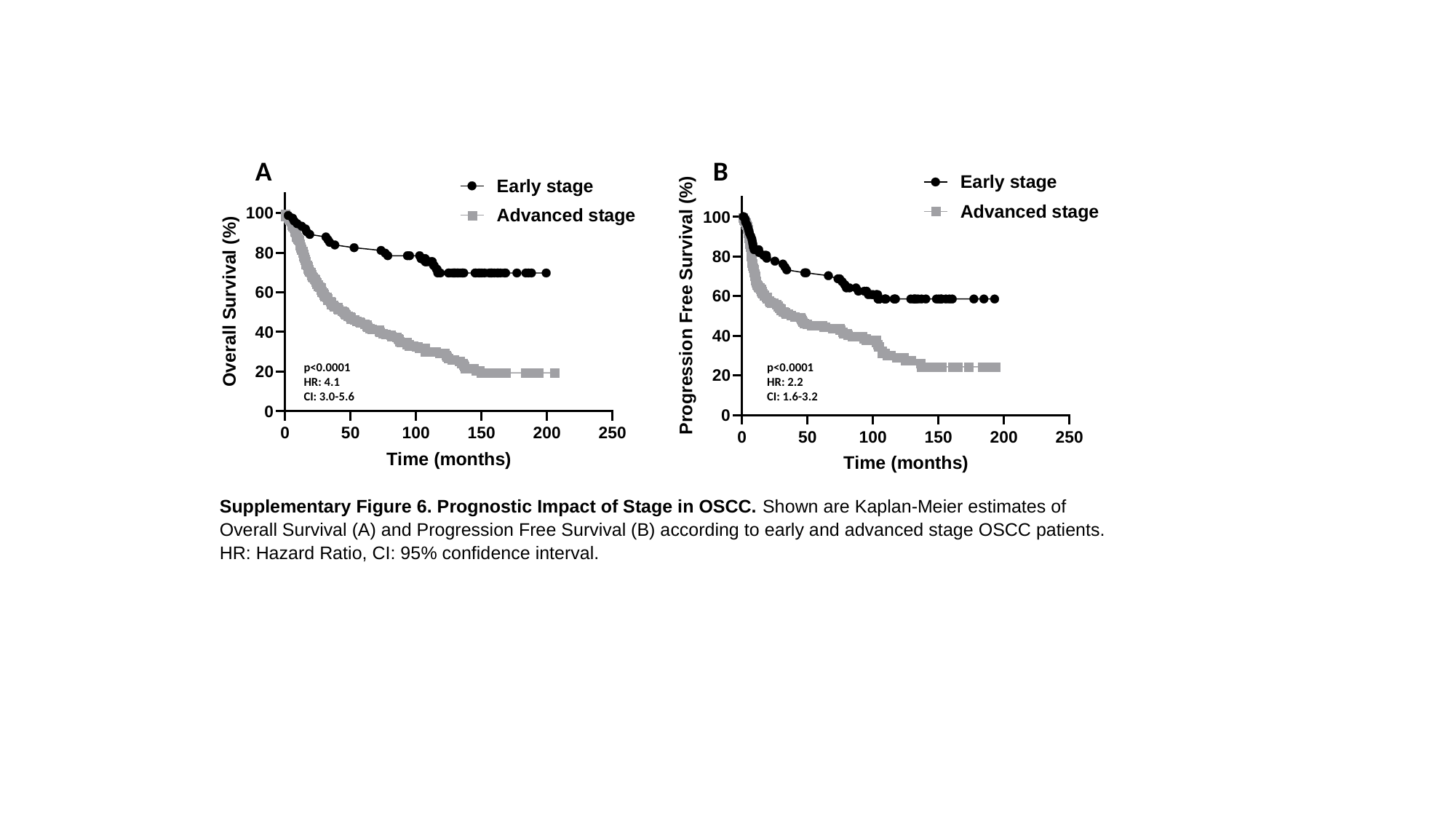

A
B
p<0.0001
HR: 2.2
CI: 1.6-3.2
p<0.0001
HR: 4.1
CI: 3.0-5.6
Supplementary Figure 6. Prognostic Impact of Stage in OSCC. Shown are Kaplan-Meier estimates of Overall Survival (A) and Progression Free Survival (B) according to early and advanced stage OSCC patients. HR: Hazard Ratio, CI: 95% confidence interval.
